# Maternal high fat diet consumption reduces liver alpha7 nicotinic cholinergic receptor expression and impairs insulin signalling in the offspring

**DOI:** 10.1038/s41598-019-56880-3

**Published:** 2020-01-08

**Authors:** S. O. Costa, C. M. Souza, P. G. Lanza, J. O. Sartori, L. M. Ignacio-Souza, T. Candreva, H. G. Rodrigues, A. S. Torsoni, M. Milanski, M. A. Torsoni

**Affiliations:** 1 Laboratory of Metabolic Disorders, School of Applied Sciences, University of Campinas, Campinas, Brazil; 2 Laboratory of Nutrients and Tissue Repair, School of Applied Sciences, University of Campinas, Campinas, Brazil; 3 Obesity and Comorbidities Research Center, University of Campinas, Campinas, Brazil

**Keywords:** Homeostasis, Type 2 diabetes, Obesity

## Abstract

The activation of nicotinic acetylcholine receptor α7 subunit (α7nAChR) has been associated to anti-inflammatory response in macrophages. High-fat diet (HFD) consumption during pregnancy and lactation impairs the cholinergic anti-inflammatory pathway in liver and white adipose tissue of offspring. In order to evaluate the relationship between damage in the cholinergic anti-inflammatory pathway and insulin resistance (IR) development, the liver of offspring of obese dams was investigated. Additionally, the capacity of α7nAChR activation to reduce IR induced by saturated fatty acid was investigated in hepatoma cell line. Initially, female mice were subjected to either standard chow (SC) or HFD during pregnancy and lactation period. After weaning, only male offspring from HFD dams (HFD-O) and SC dams (SC-O) were fed with the SC diet. Hepatic α7nAChR expression was downregulated, and hepatic TNF-α, IL-1β, and pIKK level, but not pJNK, were elevated in the HFD-O compared to SC-O mice. Besides, hepatic expression of TNF-α in response to lipopolysaccharide (LPS) was higher in HFD-O than SC-O mice. Insulin-stimulated phosphorylation of the AKT was lower in HFD-O compared to SC-O. Additionally, insulin-stimulated phosphorylation of the AKT in KOα7^Alb-Cre^ mice fed HFD was lower than WT mice fed HFD. In hepatoma cell line, palmitate increased IL-6 and TNF-α expressions and pJNK level. These effects were accompanied by reduced capacity of insulin to stimulate AKT phosphorylation. PNU or nicotine reduced cytokine expression and JNK activation, but improved insulin resistance induced by palmitate. Our results suggest that maternal obesity impairs hepatic α7nAChR expression and AKT phosphorylation in the offspring. *In vitro* studies suggest that α7nAChR activation has potential to reduce deleterious effect of saturated fatty acids on insulin signalling.

## Introduction

Cholinergic anti-inflammatory pathway activation reduces inflammatory cytokines expression through the activation of nicotinic acetylcholine receptor α7 subunit (α7nAChR) by acetylcholine^1,2^. This mechanism was first described to occur in macrophages, and it comprises an afferent arm that senses inflammation and an efferent arm that inhibits innate immune responses^1,3^. Activation of anti-inflammatory cholinergic pathway by agonist nicotine inhibits the inflammatory response, while antagonist of α7nAChR blocks the anti-inflammatory effect^4,5^. Moreover, mice α7nAChR^−/−^ produces significantly more LPS-induced TNF-α, IL-1β and other cytokines systemically, and the electrical stimulation of the vagus nerve was ineffective to attenuate LPS-induced inflammation^6^.

Macrophage activation and inflammation are elevated in obesity. Characteristics of obesity-induced inflammation include elevated production of proinflammatory molecules by adipose tissue and activation of a network of inflammatory signalling pathways. These are important factors for the development of insulin resistance^7,8^.

In previous studies, we showed that diet-induced maternal obesity leads to increased susceptibility to obesity and impairment of insulin signalling in offspring in early and late life^9^, inflammatory pathway activation^10–12^ and hypothalamic endoplasmic reticulum stress^12^. Recently, we also showed that high-fat diet (HFD) during pregnancy and lactation impairs the cholinergic anti-inflammatory pathway in liver and white adipose tissue and exacerbates cytokine production in response to LPS^11^. Therefore, it is possible that HFD could enable the expression and secretion of inflammatory cytokines and finally contribute to the development of insulin resistance in the offspring. Here, we evaluated the effect of maternal HFD consumption in the liver inflammatory response, cholinergic pathway and insulin AKT activation in the offspring recently weaned. To establish the correlation among liver α7nAChR activation, inhibition of inflammatory pathways and improvement in the insulin signalling, we used mouse hepatoma cell line treated with saturated fatty acid (SFA) in the presence or absence of pharmacological agonists of α7nAChR.

## Results

### Maternal HFD consumption reduces liver α7nAChR expression and activates inflammatory pathway

**Figure 1:**
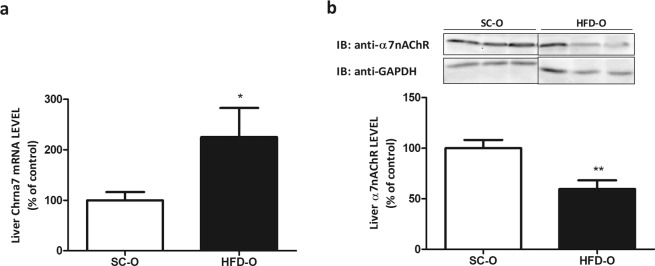
Nicotinic receptor expression in the offspring (P28). Hepatic α7nAChR mRNA (**a**) and protein levels (**b**) were evaluated by Western blot in control and obese dams’ offspring (P28). The percent expression of control (GAPDH) is shown (means ± SD, n = 6 pups per group). Black line of gel (Fig. 1b) indicates that gels/blots were cropped from different parts of the same gel. Statistical significance was analysed by Student’s t-test (*p < 0.05, **p < 0.01).

**Figure 2:**
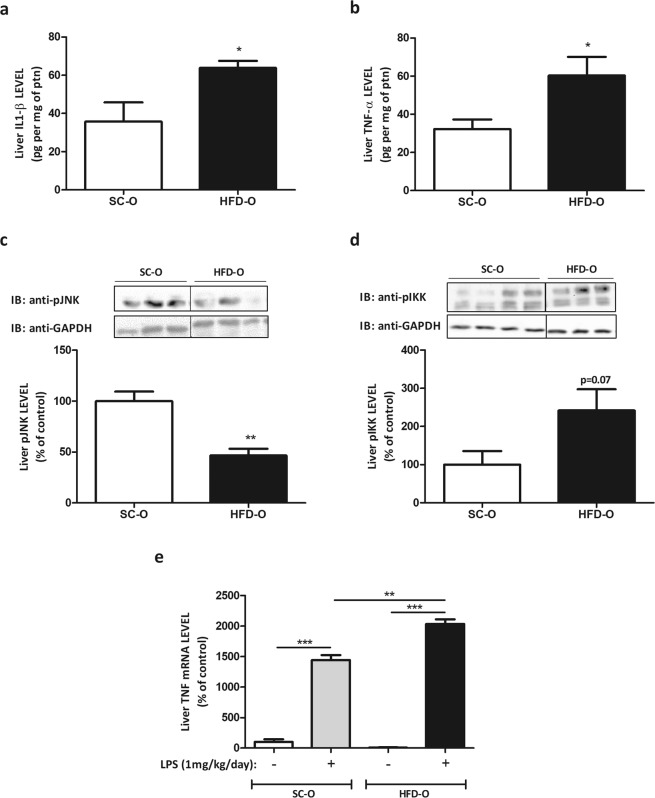
Cytokine expression in offspring’s liver. Hepatic IL1-β (**a**), TNF-α (**b**), pJNK (**c**) and pIKK (**d**) protein levels were evaluated by ELISA and Western blot in control and obese dams’ offspring (P28). The expression of the cytokines is shown per mg of protein content. Hepatic TNF-α (**e**) mRNA levels were evaluated by RT-PCR in control and obese dams’ offspring (P28) after treatment with LPS i.p. 1 mg per kg body weight for 72 hours. The percent expression of control (GAPDH) is shown (means ± SD, n = 5 pups per group). Black line of gel (Fig. 2c,d) indicates that gels/blots were cropped from different parts of the same gel. Statistical significance was analysed by ANOVA and Bonferroni post-hoc tests (*p < 0.05, **p < 0.01, ***p < 0.001), or Student’s t-test for analysis of two groups (p = 0.07).

First, we monitored the body weight gain of dams fed either standard chow (SC) or a high-fat diet (HFD). Body weight gain in the adaptation period was higher in HFD-fed than SC-fed dams (Fig. S1a). During pregnancy the body weight gain was similar for both groups (Fig. S1b) but during the suckling period (Fig. S1c) body weight gain was higher in offspring from HFD-fed dams (HFD-O) than in offspring from SC-fed dams (SC-O). Next, we evaluated the expression of α7nAChR in liver of HFD-O and SC-O mice. The mRNA level of α7nAChR was significantly higher in HFD-O than SC-O mice (2.5-fold). However, the amount of liver α7nAChR protein was diminished (1.7-fold) in HFD-O compared to SC-O mice (Fig. 1a,b). Since α7nAChR has important role in the inhibition of inflammatory cytokines expression, we evaluated the hepatic IL-1β and TNF-α levels. Both cytokines presented higher levels in HFD-O than SC-O mice (Fig. 2a,b). On the other hand, JNK phosphorylation (pJNK) in liver was significantly reduced (2.1-fold), while IKK phosphorylation (pIKK) showed a tendency to increase in HFD-O compared to SC-O mice (Fig. 2c,d). LPS injection in SC-O and HFD-O mice increased hepatic TNF-α mRNA level in both groups, but the effect was more exacerbated in HFD-O compared to SC-O mice (14- and 1.4-fold, respectively) (Fig. 2e).

### Maternal HFD consumption impairs AKT phosphorylation stimulated by insulin

**Figure 3:**
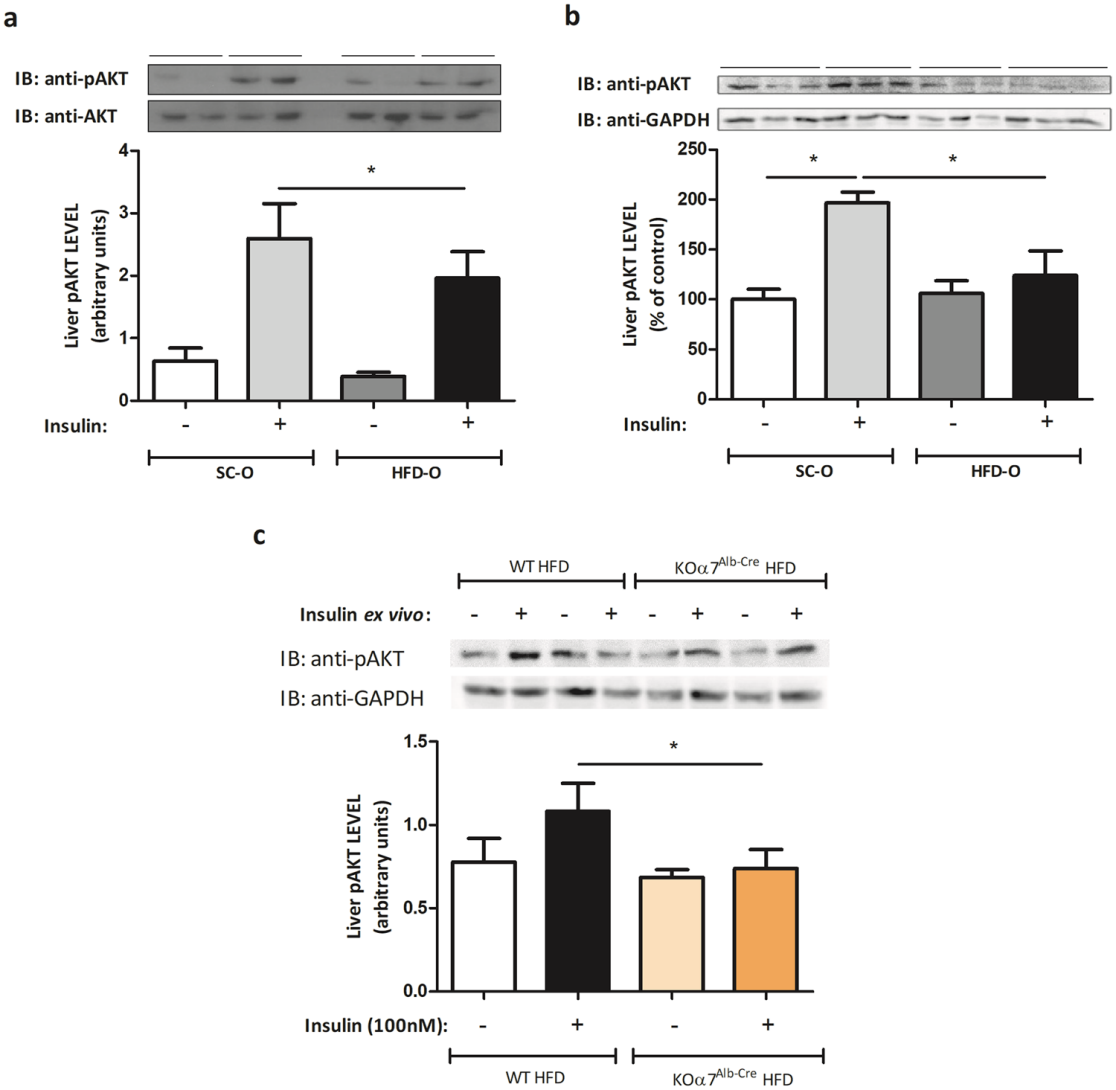
Impairment of the insulin signalling in the obese mother’s offspring. Hepatic pAKT protein levels were evaluated by Western blot in control and obese dams’ offspring (P28) after a bolus injection of saline or regular insulin (5 UI) through the abdominal cava vein. The expression of the protein was measured after 45 seconds of insulin administration. The percent expression of control (AKT) is shown (means ± SD, n = 6 per group). (**a**) Hepatic pAKT protein levels were evaluated by Western blot in control and obese dams’ offspring (P28) after an *ex vivo* experiment where the tissue was treated with insulin (100 nM) for 10 minutes in the cell media. The percent expression of control (GAPDH) is shown (means ± SD, n = 6 pups for SC-O and n = 4 pups for HFD-O). (**b**) Hepatic pAKT protein levels were evaluated by Western blot in KOα7^Alb-Cre^ and WT mice after an *ex vivo* experiment where the tissue was treated with insulin (100 nM) for 10 minutes in the cell media. The percent expression of control (GAPDH) is shown (means ± SD, n = 6 pups for SC-O and n = 4 pups for HFD-O). (**c**) Statistical significance was analysed by Student’s t-test for analysis of two groups (*p < 0.05).

AKT phosphorylation is classically affected by inflammatory pathways. To investigate insulin resistance development in the offspring of obese dams, we evaluated hepatic AKT phosphorylation stimulated by insulin using two protocols (*in vivo* and *ex-vivo*) (Fig. 3a,b). Both protocols showed that AKT phosphorylation stimulated by insulin was smaller in HFD-O than SC-O mice. Additionally, we evaluated insulin-stimulated phosphorylation of AKT in HFD-fed KOα7^Alb-Cre^ mice compared to HFD-fed WT mice. As can be observed in Fig. 3c, AKT phosphorylation was lower in KOα7^Alb-Cre^ mice than in WT mice.

### PNU and nicotine reduce inflammatory pathway activation in hepatoma cell line induced by palmitate

**Figure 4:**
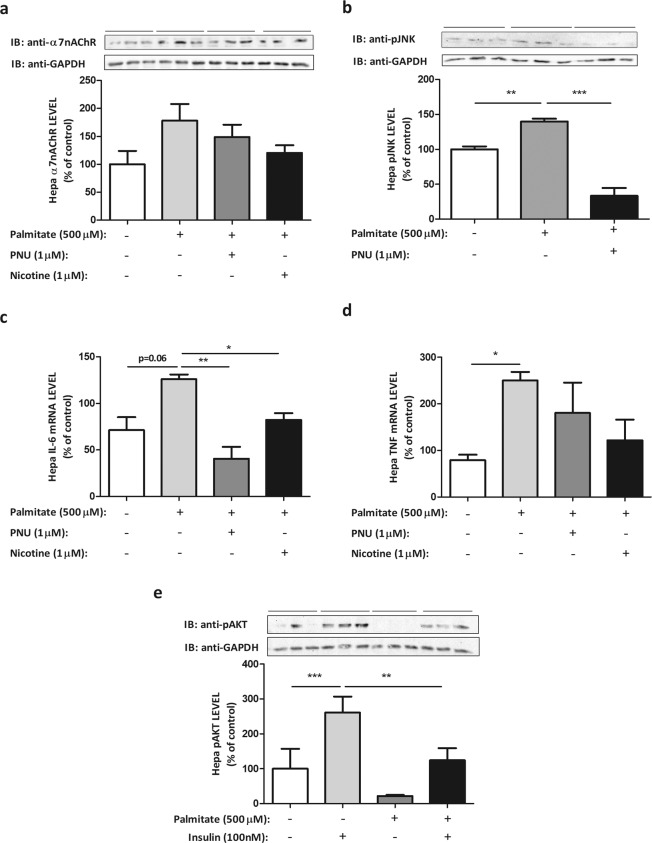
Palmitate and cholinergic agonists modulate the inflammatory pathway and insulin resistance in hepatocyte lineage. α7nAChR (**a**), pJNK (**b**) evaluated by Western blot (WB), and IL-6 (**c**) and TNF-α (**d**) mRNA levels evaluated by RT-PCR, and pAKT (**e**) evaluated by WB in hepatoma cells lineage, Hepa-1c1c7 (ATCC® CRL-2026™), after treatment with palmitate (500 µM) for 3 hours and nicotine (1 µM) or PNU (1 µM) for 15 minutes, or insulin (100 nM) for 10 minutes. The percent expression of control (GAPDH) is shown (means ± SD, n = 3 independent experiments with triplicate each). Statistical significance was analysed by ANOVA and Bonferroni post-hoc tests (*p < 0.05, **p < 0.01, ***p < 0.001).

**Figure 5:**
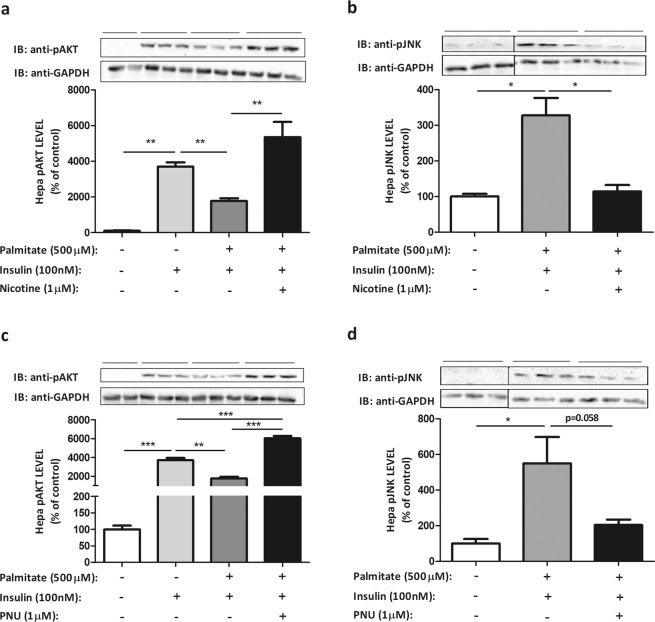
Cholinergic agonists improve the insulin signalling in hepatocyte lineage after treatment with palmitate. pAKT (**a**,**c**) and pJNK (**b**,**d**) protein levels were evaluated by Western blot in hepatoma cells lineage, Hepa-1c1c7 (ATCC® CRL-2026™), after treatment with palmitate (500 µM) for 3 hours, insulin (100 nM) for 10 minutes and nicotine (1 µM) or PNU (1 µM) for 15 minutes. The percent expression of control (GAPDH) is shown (means ± SD, n = 3 independent experiments with triplicate each). Black line of gel (Fig. 5b,d) indicates that gels/blots were cropped from different parts of the same gel. Statistical significance was analysed by ANOVA and Bonferroni post-hoc tests, or Student’s t-test for analysis of two groups (*p < 0.05, **p < 0.01, ***p < 0.001).

We characterised first the inflammatory response to palmitate of Hepa-1c1c7 cell line. As shown in Fig. 4a, cellular exposition to palmitate induced a slight increase in α7nAChR expression, but neither PNU nor nicotine changed the expression of α7nAChR significantly. To investigate the role of PNU in the activation of inflammatory pathways by the exposition to palmitate, we evaluated JNK phosphorylation (pJNK). As shown in the Fig. 4b, the exposition to palmitate increased (1.4-fold) pJNK level, but the addition of PNU reduced pJNK level significantly (76%). The level of pIKK was also investigated, but treatment with palmitate did not alter the phosphorylation significantly (data not shown). Additionally, the treatment of cells with palmitate increased TNF-α mRNA (Fig. 4d) (3.1-fold) and showed a tendency (p = 0.06) to increase the levels of IL-6 mRNA (Fig. 4c). PNU was efficient in reducing IL-6 mRNA level induced by palmitate (Fig. 4c), but to TNF-α mRNA levels, the effect was not significant. Similar results were observed in the presence of nicotine (Fig. 4c,d). AKT phosphorylation was used as a marker of the effect of inflammatory pathway on insulin signalling. As observed, insulin treatment of Hepa-1c1c7 cells increased (2.5-fold) AKT phosphorylation, but the previous treatment with palmitate reduced (52%) the capacity of insulin to stimulate AKT phosphorylation (Fig. 4e) and increased JNK phosphorylation (Fig. 5b,d). The activation of α7nAChR receptor prevented the harmful effect of palmitate on the insulin-stimulated AKT phosphorylation. As shown in Fig. 5a,c, insulin-stimulated AKT phosphorylation was increased (3.4-fold) while pJNK level was reduced (2.8-fold) in the presence of nicotine or PNU, agonists of α7nAChR receptor (Fig. 5b,d).

## Discussion

The activation of inflammatory pathways is known to induce insulin resistance in central and peripheral tissues^13–15^. Saturated fatty acids and LPS can activate TLR4 receptor and stimulate inflammatory cytokines expression, leading to activation of serine kinases (JNK and IKK) that are responsible for inhibiting insulin signalling^7,8,16^. JNK and IKK activation are also observed in diet-induce obesity (DIO) and genetic models^17,18^. In metabolic programming, maternal obesity also contributes to the activation of inflammatory pathways, hypothalamic endoplasmic reticulum stress and damage to glucose homeostasis^9–12^.

Here, we showed that recently weaned offspring mice from dams fed with HFD during pregnancy and lactation have increased hepatic concentration of inflammatory cytokines (IL1β and TNF-α) as well as phosphorylation of JNK and IKK. Moreover, liver TNF-α mRNA expression induced by LPS was more pronounced in HFD-O compared to SC-O mice. Interestingly, although α7nAChR mRNA level was increased in HFD-O compared to SC-O mice, hepatic α7nAChR protein level was reduced in HFD-O compared to SC-O mice. Since α7nAChR gene expression was raised and protein level was diminished in HFD-O mice, post-translational modifications may be acting and stimulating α7nAChR degradation. Nicotinic acetylcholine receptors are a target of the ubiquitin-proteasome system, as demonstrated in α3, α7, β2 and β4 subunits^19,20^, directing the ubiquitinated subunit for proteasomal degradation. HFD-O mice showed a slight increase in ubiquitination of α7nAChR but this was not significant. To further explore the role of the post-translational mechanism acting on the α7nAChR protein level we evaluated the expression of RIC3, an important chaperone protein that influences the folding and assembly of α7nAChR in the endoplasmic reticulum. However, RIC3 expression was no different in HFD-O compared to SC-O mice.

The receptor α7nAChR is an important component of cholinergic anti-inflammatory pathway^2,6^. Although in a previous study, we also observed the negative effect of maternal HFD consumption on the hepatic α7nAChR expression in the offspring, leading to higher susceptibility to activation of inflammatory pathway compared to SC-O mice^11^, we have not investigated the relationship with the development of insulin resistance. Here, HFD-O mice did not show difference in the basal glycaemia (data not shown), but AKT phosphorylation stimulated by insulin was reduced in HFD-O compared to SC-O mice. The present data, together with previous results showing that liver JNK phosphorylation and glucose production were increased in HFD-O, as indicated by pyruvate tolerance test (PTT) and liver PEPCK expression^12^, point to the development of insulin resistance. Although the focus of our investigation has been the liver, we showed in a previous study using the same model that white adipose tissue and soleus muscle also presented insulin resistance^9^. These effects were stable considering that insulin resistance in offspring from obese dams was detected until 82-days-old mice^9^.

Previously we showed that hepatic cyclic adenosine monophosphate (cAMP) response element binding protein (CREB) phosphorylation was reduced in HFD-O mice compared to SC-O mice^11^. CREB phosphorylation is induced by cAMP and it plays an important role in the recruitment of coactivators and activation of gluconeogenesis^21–24^. However, insulin signalling induces CREB-binding protein (CBP) phosphorylation, thus reducing the recruitment of coactivators and consequently gluconeogenesis^25^. Furthermore, CREB also participates in anti-inflammatory signalling induced by α7nAChR activation. As demonstrated by Yoshikawa and colleagues, α7nAChR activation inhibits IκB phosphorylation, NFκB transcriptional activity and reduces cytokine expression via AKT and CREB^26^. Additionally, Chiefari and colleagues, in a recent review, discussed the participation of high mobility group A1 (HMGA1) in glucose homeostasis^27^; these authors provided evidence of previous studies indicating that HMGA1, cAMP, protein kinase A (PKA) and CREB play an important role in gluconeogenesis activation. This pathway is inhibited by inactivation of HMGA1 due to insulin-induced HMGA1 phosphorylation^27^. Considering the relation between α7nAChR activation and CREB phosphorylation, HMGA1 could be an additional pathway modulated by the cholinergic receptor that has an important effect on glucose homeostasis. The development of insulin resistance in HFD-O mice may be related to diminished expression of α7nAChR and reduction of the phosphorylated CREB level in hepatocytes. In this condition, inflammatory cytokine expression can damage insulin signalling and glucose homeostasis.

Recently, in an elegant study, Li *et al*.^28^ demonstrated that HFD consumption was more harmful to α7nAChR^−/−^ than wild type (WT) mice. The HFD-fed α7nAChR^−/−^ mice showed more pronounced hepatic lipid accumulation, macrophage infiltration and mRNA levels of TNF-α, IL-6 and IL-1β than HFD-fed WT mice. In addition, liver insulin signalling was significantly damaged in HFD-fed α7nAChR^−/−^ mice compared to HFD-fed WT mice. Here, we showed that HFD-O mice has reduced expression of α7nAChR, which could predispose the mice to metabolic damages. However, differently of offspring from obese dams (HFD-O) explored in this manuscript, Li and colleagues^28^ demonstrated that HFD-fed WT mice presented increased liver expression of α7nAChR over the period fed with HFD (8 weeks), implicating α7nAChR in the development of non-alcoholic fatty liver disease (NAFLD). However, the authors showed that the specific α7nAChR activation with PNU partly rescued the NAFLD phenotypes. Thus, maternal consumption of HFD can impair liver α7nAChR expression and contribute to the early onset of inflammatory changes and homeostasis damage in the offspring. To further explore the participation of liver α7nAChR in the development of insulin resistance, we investigated insulin-stimulated phosphorylation of AKT in HFD-fed KOα7^Alb-Cre^ mice with deletion of α7nAChR in the hepatocytes. AKT phosphorylation was lower in KOα7^Alb-Cre^ than WT mice, suggesting a protective effect of α7nAChR in hepatocytes under inflammatory conditions.

Cholinergic anti-inflammatory pathway and α7nAChR activation have been associated to attenuate inflammatory response in endotoxemia^5^, macrophage TNF-α release^6^, hepatic lipid accumulation and damage to glucose homeostasis^28^. However, the effects that were associated to the activation of cholinergic receptor could have come from different cell types present in the liver. Li *et al*.^28^ used knockout mice (α7nAChR^−/−^) to evaluate the effect of HFD consumption on liver metabolic disturbances related to lipid accumulation. Although the findings are very important to relate α7nAChR to the progression of NAFLD and insulin resistance, the effects described may have arisen from α7nAChR located in hepatocytes or macrophages for example.

The controversy about inflammation triggered from interaction between saturated fatty acid and TLR4^29^ has received more information recently^30^. However, cells incubated with saturated fatty acids showed increased inflammatory markers^31,32^. Thus, the exposition of cell culture to saturated fatty acid is an excellent model to stimulate inflammatory pathway activation and induce insulin resistance. The hepatoma cell line Hepa-1c1c7 (ATCC® CRL-2026™) exposed to palmitate presented increased JNK phosphorylation, IL-6 and TNF-α mRNA compared to cells exposed to vehicle solvent alone. Moreover, these effects were accompanied by insulin resistance, as measured by reduced AKT phosphorylation stimulated by insulin in cells previously incubated with saturated fatty acid. To evaluate if α7nAChR activation could reduce the inflammation and insulin resistance, we used nicotine and PNU, which are agonist of cholinergic receptor. Both agonists were efficient to reduce the inflammatory marker levels in cells exposed to saturated fatty acid and improve insulin signalling.

In conclusion, we demonstrated that α7nAChR activation in hepatocytes is able to improve insulin signalling through inhibition of cytokines expression and JNK activation independent of α7nAChR activation in macrophages. Moreover, although HFD consumption increased liver expression of α7nAChR, as demonstrated by Li and colleagues^28^, here we showed that maternal HFD consumption diminishes hepatic α7nAChR expression, increases hepatic cytokines mRNA level and induces insulin resistance in the offspring.

## Methods

### Animals

**Figure 6:**
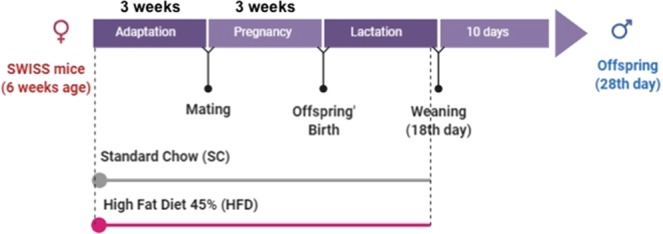
Experimental protocol of *in vivo* experiments.

Swiss female mice aged 5–6 weeks were obtained from the Multidisciplinary Center for Biological Research at University of Campinas (Campinas, Brazil). The mice were held in a temperature-controlled environment (12 h light/dark cycle). Ethics approval was obtained from the State University of Campinas Ethics Committee (Protocol 4328-1) and all experiments were performed in accordance with relevant guidelines and regulations for the care and use of laboratory animals. Dams were randomly separated in two different groups (n = 10 dams per group), fed with either high-fat diet (HFD) or standard chow diet (SC) (NUVILAB® Cr-1, Nuvital, PR-Brazil) (Table 1), ad libitum during pregnancy and lactation. After birth the litter size was adjusted to eight animals per litter. HFD was prepared in our laboratory according to the AIN-93G but modified for high-fat content (45%) as previously described^12^. Male offspring were weaned on the 18^th^ day after birth and fed with standard chow until 28^th^ day (Fig. 6). Mice with liver-specific Chrna7 deficiency were generated using the Cre-*lox*P system. To obtain KOα7^Alb-Cre^ mice we mated Chrna7^flox/flox^ (B6(Cg)-Chrna7^tm1.1Ehs^/YakelJ) and Alb-Cre (B6.Cg-Speer6-ps1^Tg(Alb-cre)21Mgn^/J) mice. Both lineages were purchased from The Jackson Laboratory. Chrna7^flox/flox^ animals were used for the control (WT). The male mice were maintained on a 45% HFD for 8 weeks.

**Table 1:** Nutritional composition of experimental and standard chow diet.

Ingredients	Standard Chow (g%)	High Fat Diet (g%)
Carbohydrates^(a)^	55.0	41.2
Net Protein^(b)^	22.5	20.8
Fat content^(c)^	4.5	23.6
Fibrous matter	8.0	5.8
Ash matter	10.0	8.6
Total	100.0	100.0

Standard Chow: NUVILAB® Cr-1, Nuvital, PR-Brazil.

(a)Starch and saccharose.

(b)Vegetal protein—from wheat and corn (added lysine and methionine).

(c)Soy oil and lard.

### Anaesthesia and tissue extraction

Mice were anaesthetised with a mixture containing ketamine (139.2 mg kg^−1^ bw), diazepam (4 mg kg^−1^ bw) and xylazine (18.4 mg kg^−1^ bw) and subsequently euthanized by decapitation for the tissue extraction. Tissue samples were frozen in liquid nitrogen and stored at −80 °C until processing. When necessary, the samples were plated on a culture plate containing cold Dulbecco’s modified Eagle medium (DMEM; Invitrogen, USA) for analysis of the insulin signalling pathway.

### Cytokine level measurements

Mice offspring were sacrificed on the 28^th^ day after an overnight fasting period. Liver samples were collected and homogenised in extraction buffer with phosphate-buffered saline (PBS) and protease inhibitor. Afterwards, the samples were centrifuged for 30 min at 25 °C for the protein extraction. The IL-1β and TNF-α cytokine levels were measured using an Elisa Duo Set Kit (R&D System, Minneapolis, MN, USA).

### Western blot analysis

Tissues were homogenised in freshly prepared ice-cold buffer (1% v/v Triton X-100, 0.1 mol/L Tris, pH 7.4, 0.1 mol/L sodium pyrophosphate, 0.1 mol/L sodium fluoride, 0.01 mol/L EDTA, 0.01 mol/L sodium vanadate, 0.002 mol/L PMSF and 0.01 mg/mL aprotinin). The supernatant protein was separated by centrifugation (10,000 × *g*) for 30 min at 4 °C, and the protein concentration was determined using Biuret dye-bleeding method. Samples were resuspended in Laemmli sample buffer and boiled for 5 min before separation by SDS-PAGE using a miniature slab gel apparatus (BioRad, Richmond, CA, USA). Electrotransfer of proteins from the gel to a nitrocellulose membrane was performed for 10 min in a transfer buffer that contained methanol and SDS. These membranes were incubated overnight at 4 °C with specific antibodies: α7nAChR (bs-1049R; Bioss Antibodies^©^), Phospo-JNK (#9255; Cell Signaling^©^), Phospo-AKT (#4060) and Phospo-IKK (#2697). Then, after washout with a Tris-buffered saline (TBS)-Tween 20 (TTBS; 10 mmol/L Tris, 150 mmol/L NaCl, 0.5% Tween 20), the nitrocellulose membranes were probed with goat peroxidase-conjugated secondary antibodies (KPL, Gaithersburg, MD, USA) for 2 hours in room temperature. Proteins were detected by chemiluminescence kit (SuperSignal West Pico Chemiluminescent Substrate, Thermo Fisher Scientific, MA, USA) and bands were evaluated by densitometry using Scion Image software (ScionCorp, MD, USA). The intensities of the bands were normalised to those of total protein or loading control (GAPDH or β-actin).

### RT-PCR analysis

Hepatic frozen tissue was homogenised in TRIzol reagent (Life Technologies Corporation, CA, USA) for RNA extraction. After 5 min at room temperature, the chloroform was added to the homogenate. Following centrifugation, the RNA phase was precipitated with isopropyl alcohol and then washed with 75 and 100% ethyl alcohol. After drying, the pellet was resuspended in ultra-pure water stored at −80 °C. RNA was quantified in NanoDrop ND-2000 (Thermo Electron, USA). Reverse transcription was performed with 3 µg of total RNA using High-Capacity cDNA Reverse Transcription kit (Life Technologies Corporation). Relative expression was determined using the Taqman detection system and primers for the following target genes: CHRNA7 (Mm01312230_m1; Thermo Fisher Scientific, USA), IL6 (146868493; Integrated DNA Technologies-IDT, USA), TNFα (Mm00443258_m1; Thermo Fisher Scientific, USA), IL1β (Mm00434228_m1; Thermo Fisher Scientific, USA). GAPDH (4351309; Applied Biosystems, USA) was used as endogenous control. Real time PCR was performed on an AB/Prism 7500 fast platform. Data were analysed using the sequence detection system 2.0.5.

### AKT phosphorylation stimulated by insulin

#### *Ex vivo* analysis

Male mice offspring were euthanized to collect liver tissue samples as described previously. Fragments of hepatic tissue were incubated at 37 °C for 2–4 hours in 24-well culture plates containing DMEM low glucose (Invitrogen, USA). Next, the medium was removed, and the tissue washed with 1X PBS. Tissue fragments were incubated at 37 °C for 10 minutes in 24-well culture plates containing 100 nM insulin in DMEM low glucose (Invitrogen, USA). Tissues were then collected, froze in liquid nitrogen and stored at −80 °C until processing for Western blot analysis.

#### *In vivo* analysis

To evaluate AKT phosphorylation *in vivo*, a bolus injection of saline or 5 UI regular insulin (Humulin, Eli Lilly and Company, USA) was administered through the abdominal cava vein. Fragments of liver were extracted 45 seconds after insulin/saline injection. For animals stimulated with insulin, the delta value (value after stimulation − value before stimulation) was considered for the statistical analysis. Tissue samples were frozen in liquid nitrogen and stored at −80 °C until processing.

### LPS treatment

To induce inflammatory response, mice were treated with LPS diluted in sterile saline and administered intraperitoneally (IP) once a day for three days (1 mg kg^−1^ bw - IP). Mice were euthanized 2 hours after LPS treatment, and fragments of liver were collected, froze in liquid nitrogen and stored at −80 °C until processing.

### *In vitro* experiments

Hepatoma cell line, Hepa-1c1c7 (ATCC® CRL-2026™), derived from mice was used to evaluate the ability of the cholinergic pathway to modulate AKT phosphorylation induced by insulin. Cells were cultivated in alpha modified Eagle’s medium (αMEM; Invitrogen, USA) supplemented with 10% foetal bovine serum (Invitrogen, USA) and 1% penicillin (100 U/mL)/streptomycin (100 µg/mL) (Invitrogen, USA) at 37 °C and 5% CO_2_.

Cells were treated with 500 µM palmitate (palmitic acid from Sigma-Aldrich at 500 μM was first diluted in NaOH conjugated to BSA (3:1) for 45 minutes at 37 °C) for 3 hours in 6-well culture plate. The protein content was extracted and analysed by Western blotting. When necessary, 1 µM PNU-282987 (P6499-10MG; Sigma-Aldrich, Brazil) or 1 µM nicotine (N0267-100MG; Sigma-Aldrich, Brazil) was added to the medium for 15 minutes after palmitate treatment. To evaluate the insulin signalling, cells were treated with 100 nM insulin (Humulin, Eli Lilly and Company, USA) for 10 minutes after the 3 hours of palmitate treatment.

### Data presentation and statistical analysis

All results are presented as the mean ± SD. Student’s *t*-test of unpaired samples and analysis of variance (ANOVA) for multiple comparisons were carried out after confirmation of normal distribution using the Kolmogorov–Smirnov test. ANOVA was followed by the Bonferroni post-hoc test and used when differences among more than two groups were analysed. Statistical significance for all analyses was set at *p* < 0.05. All statistical comparisons were performed using GraphPad Prism 6.01 software (http://www.graphpad.com/scientific-software/prism/).

## Supplementary information

Supplementary information.
