# Supplementary material for: Maternal high fat diet consumption reduces liver alpha7 nicotinic cholinergic receptor expression and impairs insulin signalling in the offspring
Source: Sci Rep. 2020 Jan 8;10:48. doi: 10.1038/s41598-019-56880-3 (PMC6949221; doi:10.1038/s41598-019-56880-3)
Supplement: Supplementary file 1 — Supplementary information. [file 41598_2019_56880_MOESM1_ESM.docx]

Maternal high fat diet consumption reduces liver alpha7 nicotinic cholinergic receptor expression and impairs insulin signalling in the offspring

Costa, S.O.^1^; Souza, C.M.^1^; Lanza, P.G.^1^; Sartori, J.^1^; Ignacio-Souza, L.M.^1,3^; Candreva, T.^2^.; Rodrigues, H.G.^2^; Torsoni, A.S.^1,3^; Milanski, M.^1,3^; Torsoni, M.A.^1,3^*****

**Supplementary Methods**

**Immunoprecipitation analysis**

Tissues were homogenised in freshly prepared ice-cold buffer and the supernatant protein was separated by centrifugation for 30 min at 4°C, and the protein concentration was determined using Biuret dye-bleeding method, as previously described. Samples were incubated overnight at 4° C with specific antibody: α7nAChR (sc-58607; Santa Cruz Biotechnology^©^). Then, the samples were incubated with the protein A Sepharose (17-0469-01; GE Healthcare^©^) for 2 hours at 4° C. The pellet was separated by centrifugation (12,000 x *g*) for 15 min at 4°C and washed with ice-cold buffer (0,5% v/v Triton X-100; 0.1 mol/L Tris, pH 7.4; 0.01 mol/L EDTA, 0.02 mol/L sodium orthovanadate). The samples were resuspended in Laemmli sample buffer and boiled for 5 min at 95° C. Electrotransfer of proteins from the gel to a nitrocellulose membrane and western blotting analysis were performed as previously described. These membranes were incubated with specific primary antibodies: ubiquitin (sc-9133; Santa Cruz Biotechnology^©^) and α7nAChR (sc-58607; Santa Cruz Biotechnology^©^).


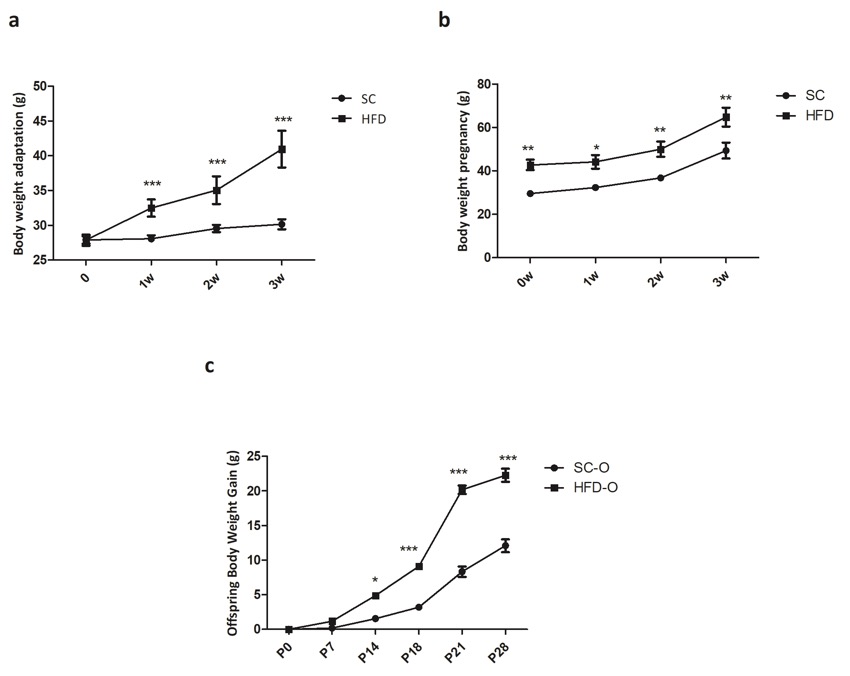


**Figure S1. Metabolic parameters of dams and offsprings.** Control and obese dams body weight gain during adaptation (**a**) and pregnancy (**b**) period. Control and obese dams’ offspring body weight gain until 28^th^ day of age (means ± SD, n=10 dams per group) (**c**). Statistical significance was analysed by ANOVA and Bonferroni post-hoc tests (*p<0.05, **p<0.01)


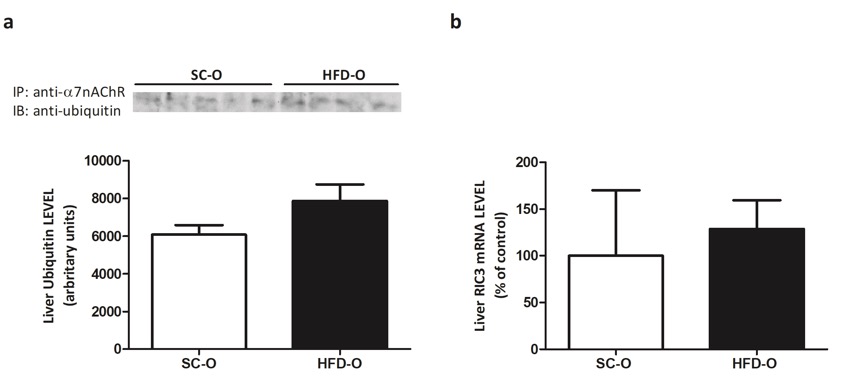


**Figure S2.** Liver co-immunoprecipitation of α7NAChR/ubiquitin (means ± SD, n=6 pups for SC-O and n=4 pups for HFD-O) and ubiquitin protein level was evaluated by Western blot (**a**) and RIC-3 mRNA level (means ± SD, n=6 pups per group) was evaluated by RT-PCR (**b**) in control and obese dams’ offspring (P28). Statistical significance was analysed by Student’s t-test (*p<0.05)
